# High Pathogenicity of a Chinese NADC34-like PRRSV on Pigs

**DOI:** 10.1128/spectrum.01541-22

**Published:** 2022-06-29

**Authors:** Lili Yuan, Zhenbang Zhu, Juan Fan, Panrao Liu, Yanhua Li, Qun Li, Zhe Sun, Xiuling Yu, Hu Suk Lee, Kegong Tian, Xiangdong Li

**Affiliations:** a Jiangsu Co-innovation Center for Prevention and Control of Important Animal Infectious Diseases and Zoonoses, College of Veterinary Medicine, Yangzhou University, Yangzhou, China; b Joint International Research Laboratory of Agriculture and Agri-Product Safety, the Ministry of Education of China, Yangzhou University, Yangzhou, China; c Yangzhou Uni-Bio Pharmaceutical Co., Ltd., Yangzhou, China; d National Research Center for Veterinary Medicine, Luoyang, China; e International Livestock Research Institute (ILRI), Hanoi, Vietnam; Changchun Veterinary Research Institute

**Keywords:** PRRSV, NADC34-like, pathogenicity, porcine reproductive and respiratory syndrome virus, viral pathogenesis

## Abstract

NADC34-like porcine reproductive and respiratory syndrome virus (PRRSV) has been reported to be prevalent in China since 2018 and became one of the main epidemic strains in some areas of China. Yet, the pathogenicity of NADC34-like PRRSV tested by experimental infection has seldomly been investigated. In this study, we infected pigs with JS2021NADC34 PRRSV, a Chinese NADC34-like PRRSV isolated in Jiangsu province in 2021, to study the pathogenicity of this virus strain. Pigs infected with this virus had lasting fever and reduced body weight with high morbidity and mortality. Histopathological changes, including interstitial pneumonia, lymphocyte depletion, acute hemorrhage, and infiltration of neutrophils in the lymphoid tissues, were observed with the viral proteins detected by immunohistochemistry staining using PRRSV-specific antibody. These results suggested that JS2021NADC34 PRRSV is highly pathogenic to pigs. As it is the latest emerging PRRSV strain in China, the prevalence and pathogenicity of NADC34-like PRRSV need to be further investigated.

**IMPORTANCE** NADC34 PRRSV was initially reported in the United States in 2018. Subsequently, this virus strain spread to other countries, including Peru, South Korea, and China. The virus was first found circulating in Northeast China and then spread to more than 10 provinces in China. NADC34 PRRSV causes severe abortion of sows and high mortality of piglets, which lead to huge economic losses to the Chinese pig industry. However, the pathogenicity of NADC34 PRRSV was rarely experimentally evaluated on pigs. In this study, pigs were infected with JS2021NADC34 PRRSV, a Chinese NADC34-like PRRSV isolated in Jiangsu province in 2021. The infected pigs had lasting fever and reduced body weight with high morbidity and mortality. Interstitial pneumonia, lymphocyte depletion, acute hemorrhage, and infiltration of neutrophils were observed in the lymphoid tissues, and high virus load was proved by immunohistochemistry staining. The above results indicated that NADC34 PRRSV has high pathogenicity on pigs.

## INTRODUCTION

Porcine reproductive and respiratory syndrome (PRRS) is one of notorious pig diseases worldwide that lead to huge economic losses to the swine industry. The etiological agent of PRRS is porcine reproductive and respiratory syndrome virus (PRRSV), which belongs to the order *Nidovirales*, family *Arteriviridae*. PRRSVs are classified into two genotypes, namely, PRRSV-1 (European type) and PRRSV-2 (North American type). According to phylogenetic analysis of ORF5 sequences, PRRSV-2 could be further divided into 9 sublineages (1). Among them, lineages 1, 3, 5, and 8 are currently circulating in China (2). Since the first report of PRRSV in 1990s, the dominant lineages of PRRSV in China have shifted from sublineage 8.7 (classical and highly pathogenic PRRSVs) to sublineage 1.8 (NADC30-like PRRSV) (2). Most recently, NADC34-like PRRSV, having a 100-amino acid (aa) deletion corresponding to positions 328 to 427 of NSP2 gene of VR-2332, was frequently reported in China (3).

The first NADC34-like PRRSV in China was reported in 2018 (3). The isolate had the highest genomic similarity with previously reported IA/2014/NADC34 strain (abbreviated as NADC34 in this study; GenBank accession no. MF326985) and therefore was designated NADC34-like PRRSV (3, 4). Since then, dozens of NADC34-like PRRSVs have been reported (5–8). In these reports, sows suffer abortion with abortion rates between 10% and 30%, and piglets have increased mortality rates ranging from 10% to 80% (2).

Although causing big problems under clinical situations, the pathogenicity of NADC34-like PRRSV was seldomly tested by experimental infection. Song et al. reported that HLJDZD32-1901, an NADC34-like PRRSV, was mildly pathogenic in piglets (6). In contrast, another Chinese NADC34-like PRRSV was reported to have high pathogenicity to pigs (9). In our previous study, we successfully isolated an NADC34-like PRRSV, designated JS2021NADC34, from a pig farm in Jiangsu province where sows suffered increased abortions and piglets had high mortality (8). Therefore, in this study, we tested the pathogenicity of this virus on pigs by experimental infection.

## RESULTS

### JS2021NADC34 PRRSV replicated efficiently in PAMs with high virus titer.

**FIG 1:**
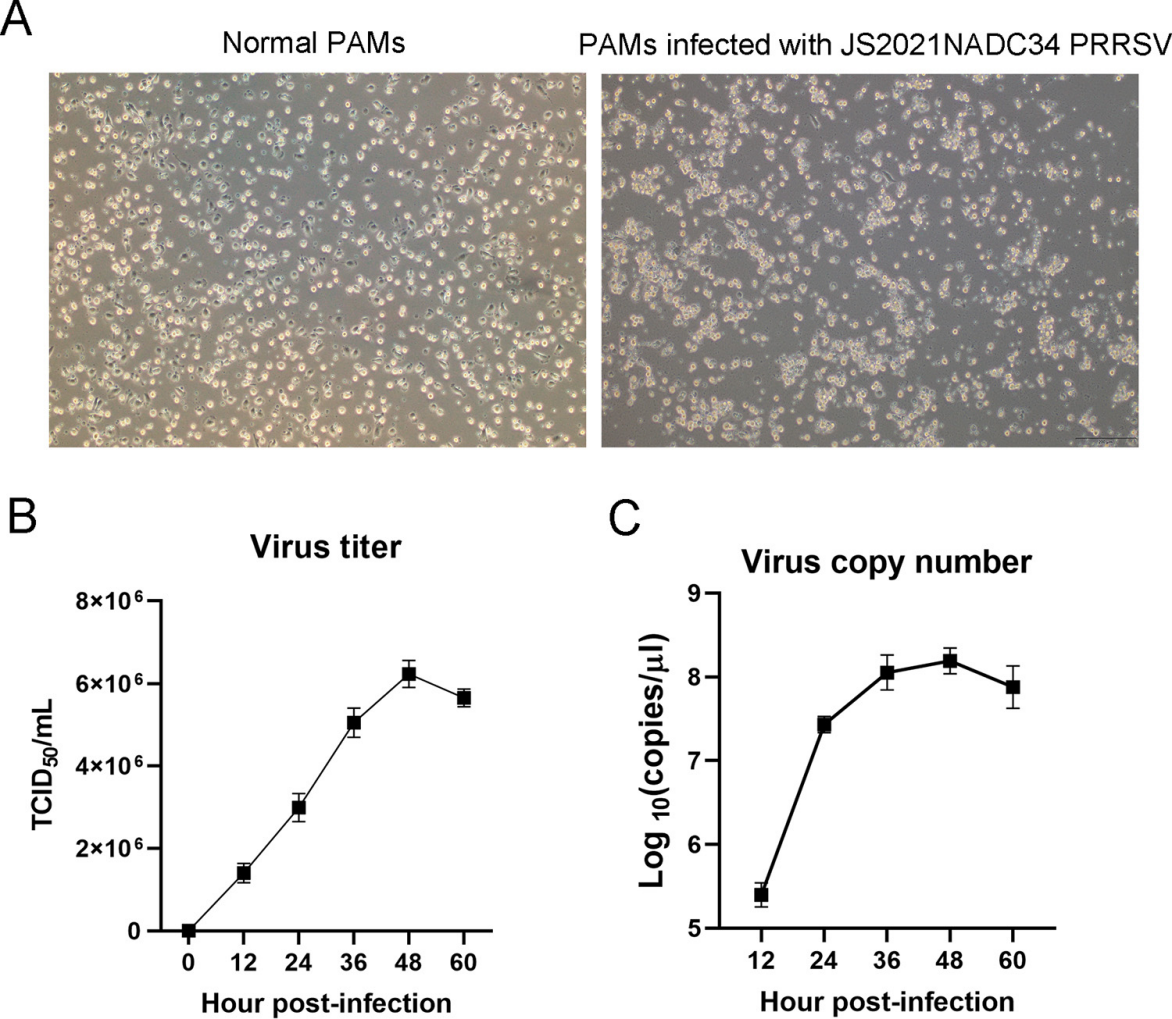
JS2021NADC34 PRRSV infection and proliferation in PAMs. (A) CPEs were observed at 24 hpi using the optical microscope in the light field (bar = 200 μM). (B and C) PAMs were infected with JS2021NADC34 PRRSV at an MOI of 1. The viral supernatants were collected from infected cells at the indicated time points and titrated by TCID_50_ (B) and quantitative RT-PCR (C). All tests were performed in triplicate and repeated twice.

JS2021NADC34 strain was previously isolated from the lung samples of disease pigs and purified by plaque assay for three rounds (8). Compared to other reported Chinese NADC34-like PRRSVs, the virus could replicate in porcine alveolar macrophages (PAMs) with apparent cytopathic effect (CPE) (Fig. 1A) but not in Marc-145 cells (data not shown). The highest virus titer of JS2021NADC34 in PAMs was 6.46 × 10^6^ 50% tissue culture infective dose (TCID_50_)/mL at 48 h postinfection (hpi) (Fig. 1B and C). The virus titer dropped to below 5.55 × 10^6^ TCID_50_/mL at 60 hpi. Therefore, the virus was harvested at 48 days postinfection (dpi) for further use.

### Genomic character of JS2021NADC34 PRRSV.

**FIG 2:**
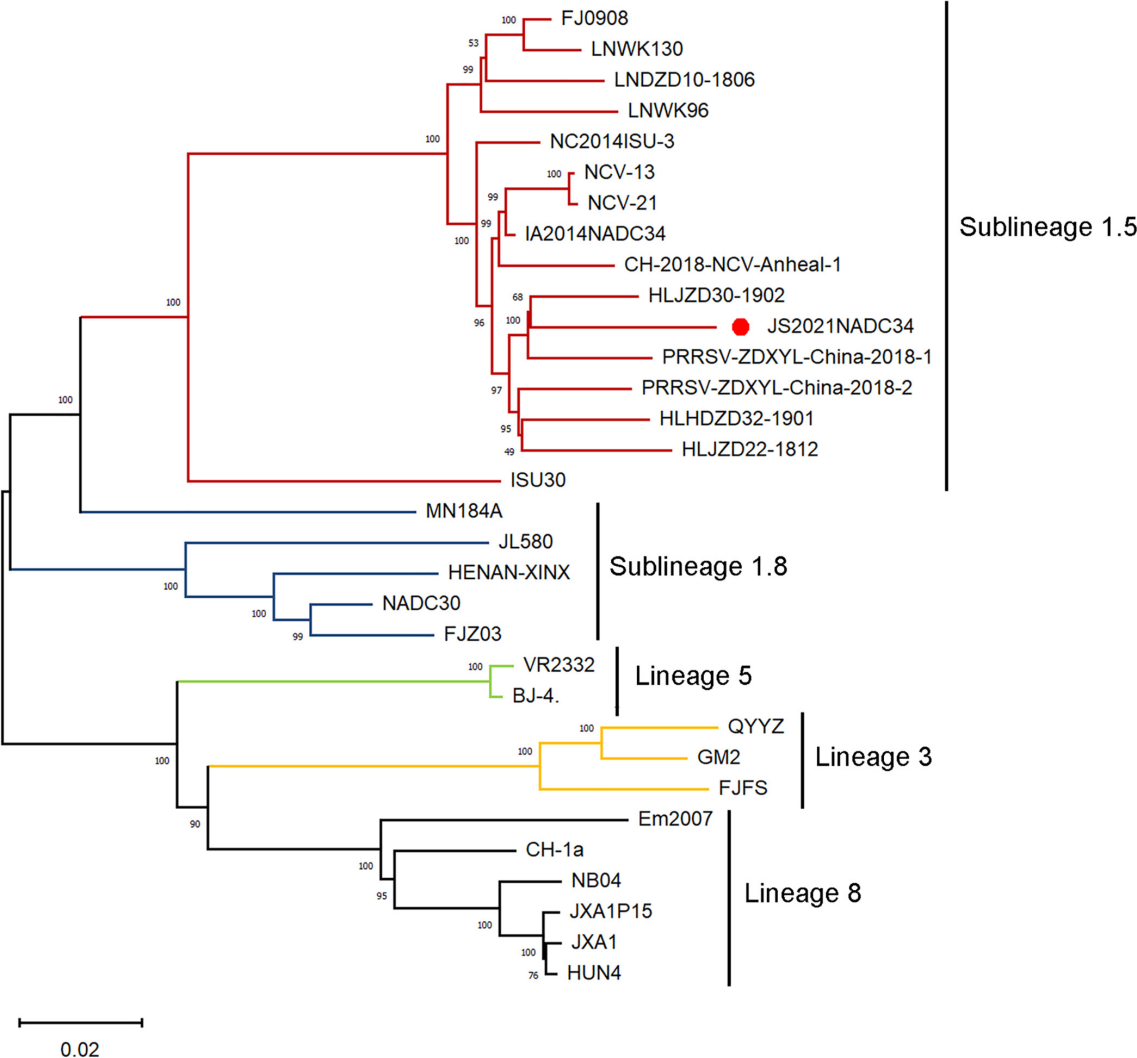
Phylogenetic analysis of JS2021NADC34 PRRSV and other reference viruses based on viral ORF5 genes. JS2021NADC34 strain was classified into sublineage 1.5. The phylogenetic tree was constructed by the distance-based neighbor-joining method using Mega-X with 1,000 replicates. The isolate JS2021NADC34 is marked with a red dot.

The full-length genome of JS2021NADC34 (GenBank access no. MZ820388) is 15,110 nucleotides long excluding the 3′poly(A) tail. Similar to other reported Chinese NADC34-like PRRSVs, JS2021NADC34 has a unique continuous 100-aa deletion in viral NSP2. Phylogenetically, it belongs to PRRSV sublineage 1.5 (Fig. 2). It shares 96.1%, 85.2%, 81.5%, 83.4%, 83.0%, and 83.2% genomic sequence identity with IA/2014/NADC34 (sublineage 1.5), NADC30 (sublineage 1.8), QYYZ (lineage 3), VR-2332 (sublineage 5.1), CH-1a (sublineage 8.1), and JXA1 (sublineage 8.3), respectively.

### Clinical presentations of pigs after JS2021NADC34 PRRSV infection.

**FIG 3:**
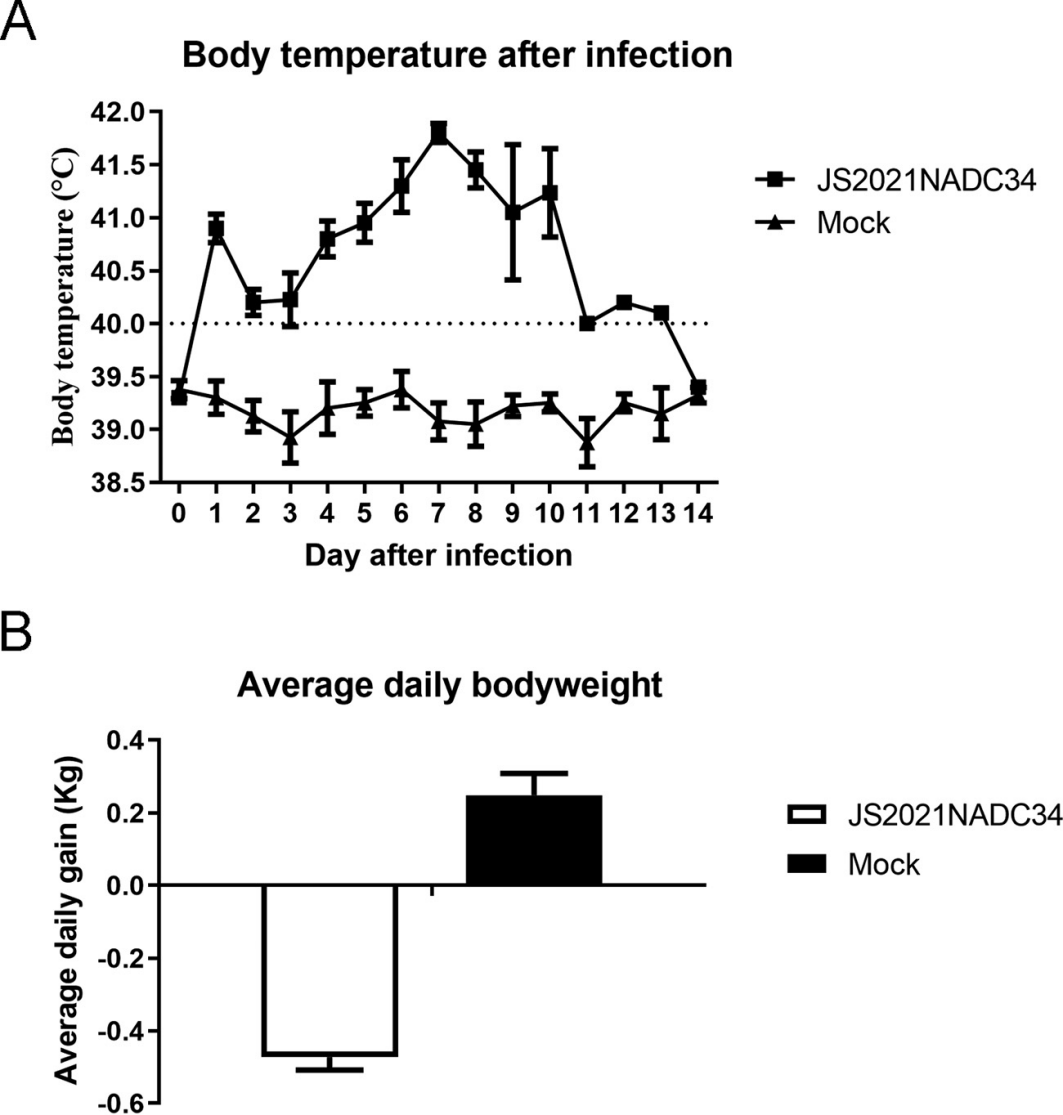
Body temperature (A) and body weight gain (B) of pigs in two groups. The fever was set as above 40.0°C. Raw data for body temperature and body weight gain were calculated and averaged.

The pathogenicity of reported NADC34-like PRRSVs differs greatly, with mortality rates ranging from 0% (0/5 pigs) to 14.3% (2/14 pigs) due to the different origins (4, 6). To test the pathogenicity of JS2021NADC34 PRRSV, 8 2-month-old pigs were randomly divided into two groups with 4 pigs in each group. Pigs in one group were infected with JS2021NADC34 PRRSV at 3 × 10^6^ TCID_50_/pig via intranasal (0.5 mL/nasal) and intramuscular (2 mL) routes simultaneously. Pigs in the control group received Dulbecco modified Eagle medium (DMEM) as a placebo. After virus infection, the pigs in the infected group showed high body temperatures above 40.5°C at 1 dpi (Fig. 3A). The body temperatures of the infected pigs dropped slightly in the next few days and returned above 41°C until 10 dpi. Two infected pigs were euthanized due to their moribund conditions at 8 dpi and one was found dead at 10 dpi. The last pig in the infected group survived and was euthanized at the end of the study, which was terminated at 14 dpi. The body temperatures of pigs in control group were normal throughout the study.

**FIG 4:**
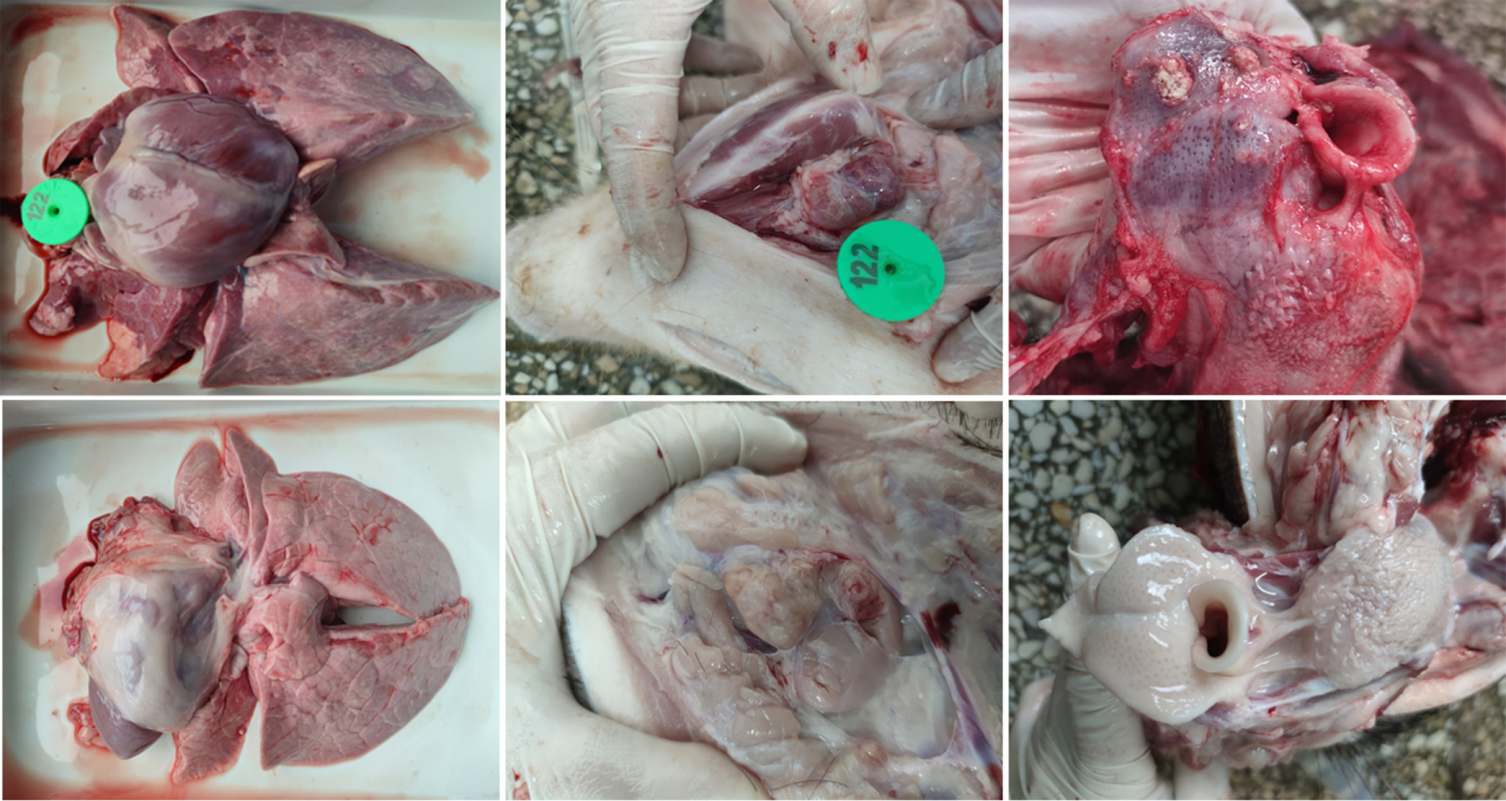
Gross pathology of lung, lymph node, and tonsil of pigs. In the upper row, severe pulmonary consolidation and necrosis were observed in the lung samples of pigs infected with JS2021NADC34 PRRSV. Hemorrhage and necrosis in the lymph node and tonsil were also observed. In the lower row, normal lung, lymph node, and tonsil of pigs in control group.

The daily body weight gain of pigs was calculated as described previously (10). As shown in Fig. 3B, the JS2021NADC34 PRRSV-infected pigs lost more than 0.4 kg of body weight per day. In contrast, the mock-infected pigs had more than 0.2 kg of body weight gain per day. Clinically, JS2021NADC34 PRRSV-infected pigs showed dehydration, respiratory distress, and shivering. At necropsy, the infected pigs had severe pulmonary consolidation and necrosis in the lung and hemorrhage and necrosis in the tonsil and lymph nodes (Fig. 4, upper row). In contrast, the above-described tissues of pigs in the control group looked normal (Fig. 4, lower row).

### Histopathological and immunohistochemistry examinations.

**FIG 5:**
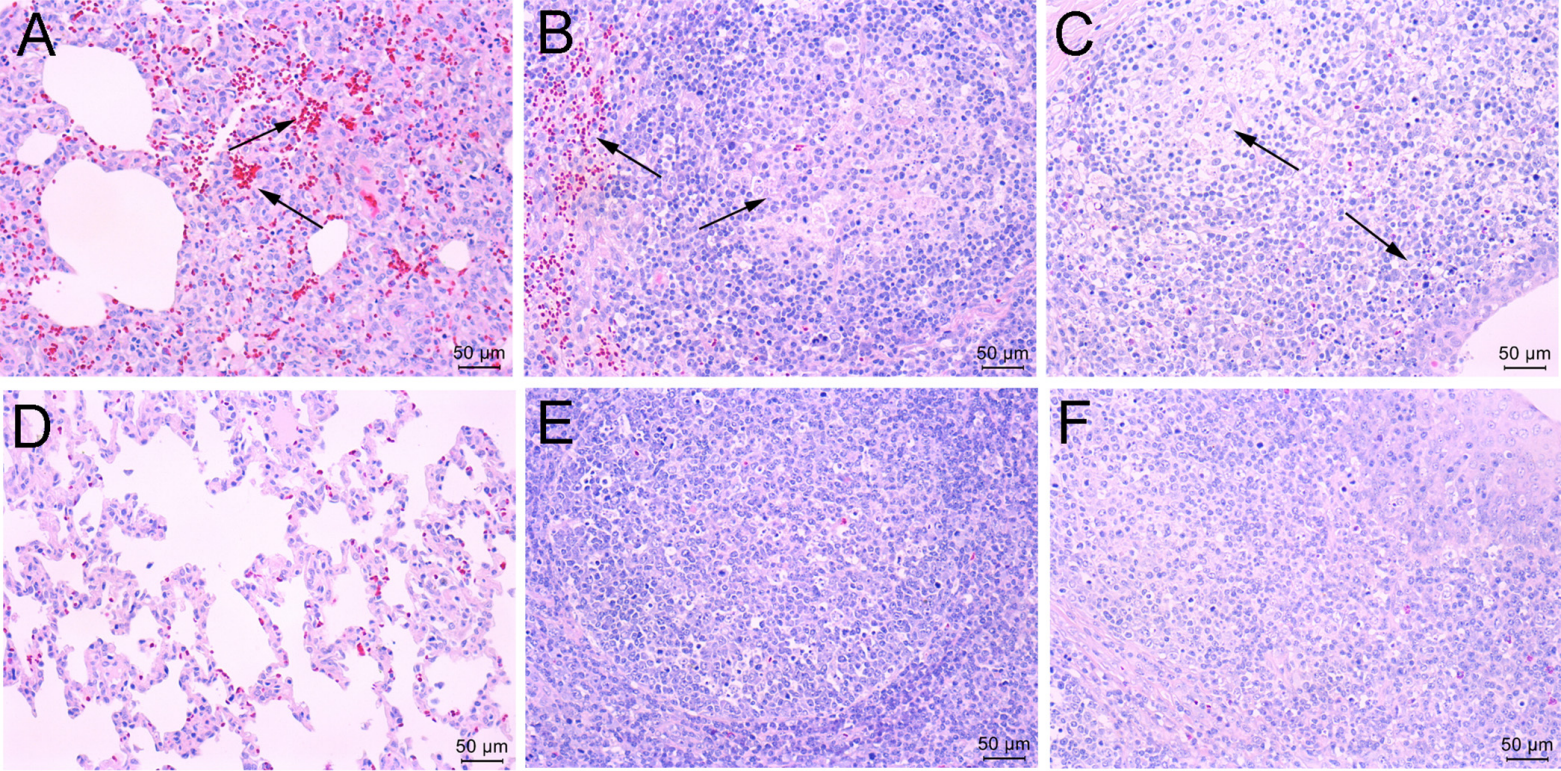
Typical histopathological manifestations in JS2021NADC34 PRRSV-infected pigs (upper row) and mock-infected pigs (lower row). (A) Lung: acute hemorrhage and interstitial pneumonia with infiltration of mononuclear cells. (B) Lymph node: vascular dilatation and lymphatic tissue necrosis with lymphocyte depletion and infiltration of neutrophils. (C) Tonsil: tonsillar lymphoid tissue necrosis and formation of large necrotic foci. (D to F) Normal lung, lymph node, and tonsil samples from mock-infected pigs. Original magnification, ×200.

Histopathological examination was next performed to evaluate the tissue damage caused by viral infection. As shown in Fig. 5, interstitial pneumonia associated with hemorrhage, which was characterized by thickening of alveolar septa, and infiltration of mononuclear cells were observed in JS2021NADC34 PRRSV-infected pigs. Lymphocyte depletion, acute hemorrhage, and infiltration of neutrophils were observed in lymph nodes and tonsils. No pathological lesions were observed in the above-described tissues of pigs in the control group.

**FIG 6:**
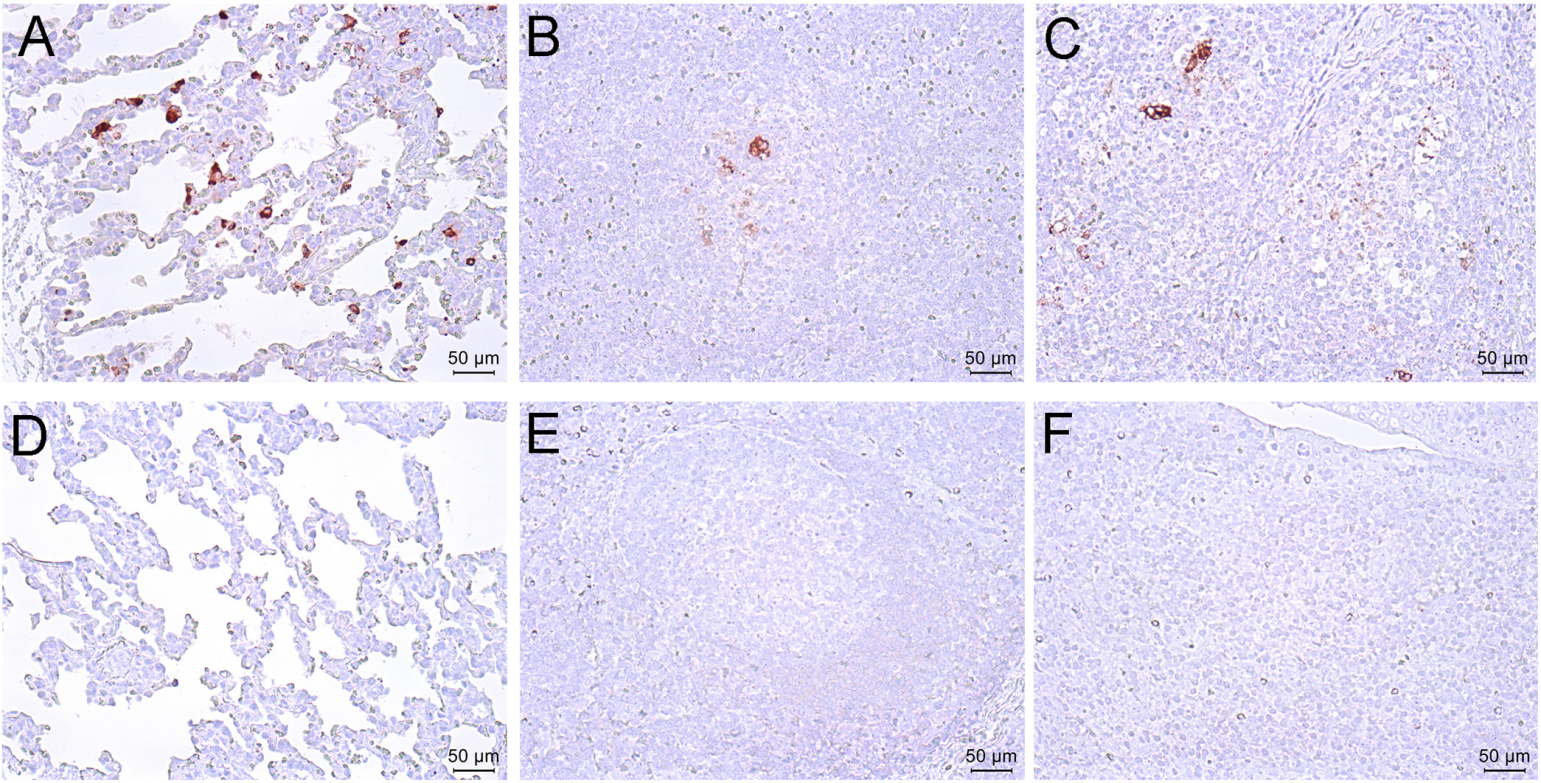
Typical IHC manifestations in the lung (A), lymph node (B), and tonsil (C) of JS2021NADC34 PRRSV-infected pigs and mock-infected pigs (D to F). Original magnification, ×200.

Monoclonal antibody specific to nucleocapsid protein of PRRSV was used in immunohistochemistry (IHC) staining to reveal the presence of viral antigen in the pig tissues. As shown in Fig. 6, positively stained epithelial cells and macrophages were observed in the lung, tonsil, and lymphoid samples of pigs infected with JS2021NADC34 PRRSV. In contrast, no positive staining was observed in the above-described tissues of pigs in control group.

### Viremia examination and serological test.

**FIG 7:**
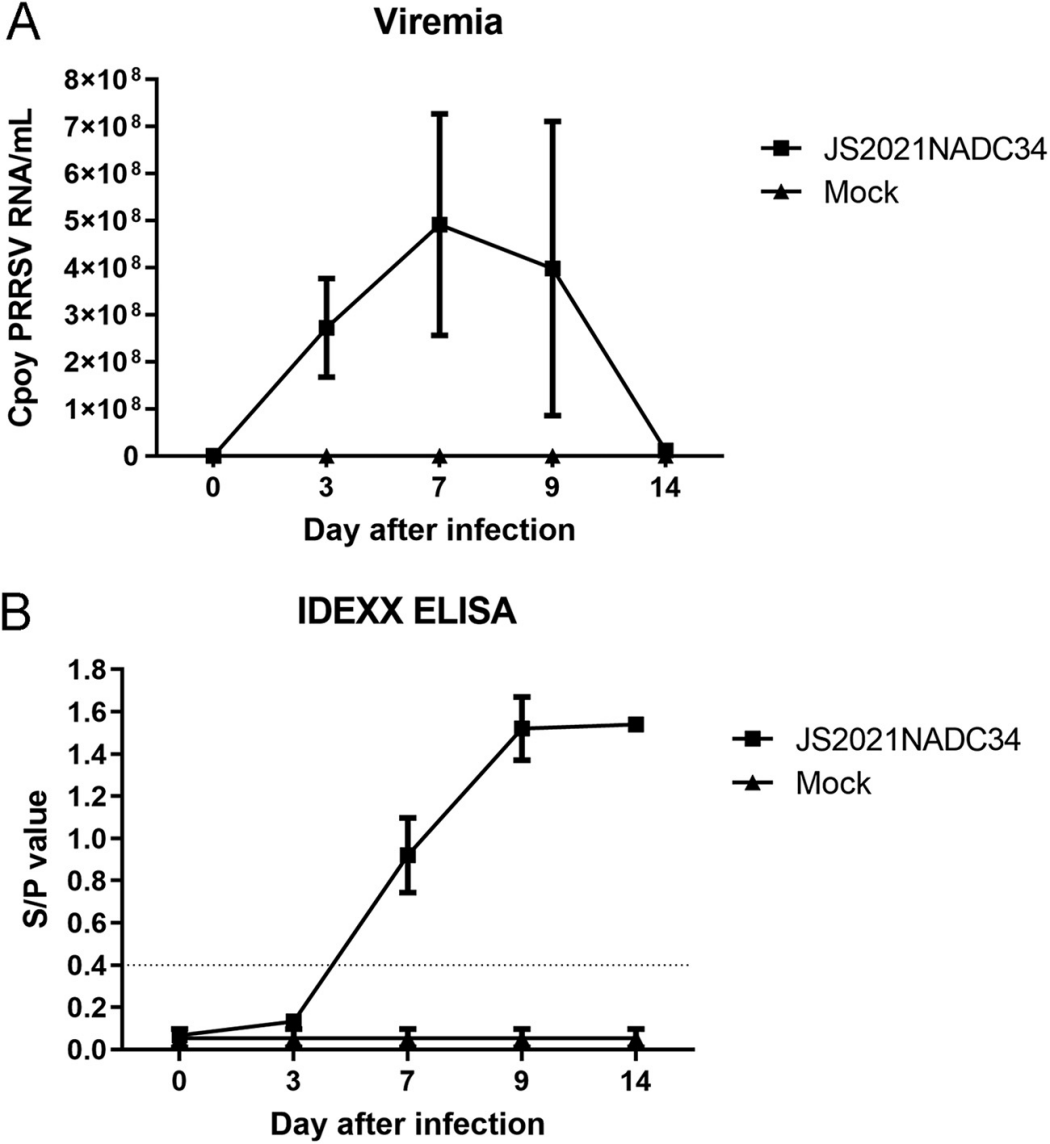
Profiles of viremia (A) and PRRSV-specific antibodies (B) in pigs. PRRSV viral RNA in serum was determined by qPCR. Pig serum was assayed for PRRSV-specific antibodies using IDEXX HerdCheck ELISA. The threshold for seroconversion was set at a sample-to-positive (S/P) ratio of 0.4 according to the manufacturer’s instructions.

Pig serum samples were collected at 3, 7, 9, and 14 dpi for viremia examination. As shown in Fig. 7A, viremia could be detected at 3 dpi and reached maximum at 7 dpi. After that, viremia of pigs decreased slightly, and viremia of one survived pig dropped to 1.19 × 10^7^ copies PRRSV RNA/mL at 14 dpi. As for the pigs in control group, no viremia was detected as expected.

PRRSV-specific antibodies after viral infection were measured using a commercial IDEXX enzyme-linked immunosorbent assay (ELISA) kit. As shown in Fig. 7B, all pigs in JS2021NADC34 PRRSV-infected group had PRRSV-specific positive antibodies at 7 dpi. The antibody titers of survived pigs in this group kept increasing until 14 dpi. As expected, no PRRSV antibodies were detected in pigs in the control group.

## DISCUSSION

Since 2013, a highly virulent ORF5 restriction fragment length polymorphism (RFLP) 1-7-4 lineage of PRRSV has emerged and caused severe abortion of sows and high mortality of piglets (4). The ORF5 RFLP pattern of 1-7-4 refers to 1 (MluI = 0 site), 7 (HindII = nucleotides [nt] 88, 219, 360), and 4 (SacII = nt 24, 555). van Geelen et al. described several isolates of the 1-7-4 lineage of PRRSV with different pathogenicity between 2013 and 2014 in different states of the Unites States (4). Among these viruses, IA/2014/NADC34 (NADC34) PRRSV was reported to be responsible for the dramatic abortion storms in sows and high mortality rates in piglets in at least five states in the United States since 2016 (4). The 1-7-4 lineage of PRRSV shares the genetic feature of a 100-aa deletion corresponding to positions 328 to 427 of the NSP2 gene of VR-2332. In addition to the United States, some other countries, including Peru and South Korea, have recently reported the prevalence of the 1-7-4 lineage of PRRSVs (11, 12).

In China, one 1-7-4 lineage of PRRSV was isolated in 2018 and was designated NADC34-like PRRSV due to the highest genomic similarity with IA/2014/NADC34 (NADC34) (3). Since then, NADC34-like PRRSVs have spread to at least nine provinces, including Heilongjiang, Jilin, Liaoning, Hebei, Henan, Shandong, Jiangsu, Sichuan, and Fujian (13, 14). Song et al. evaluated the pathogenicity of a Chinese NADC34-like PRRSV (6). In their study, pigs infected with HLJDZD32-1901, an NADC34-like PRRSV isolated in Heilongjiang in 2019, displayed mild clinical signs, including mild cough and anorexia without fever (below 39.5°C), and death. The results indicated that Chinese NADC34-like PRRSV HLJDZD32-1901 is a mildly pathogenic strain in piglets. Compared to the animal study results performed by van Geelen et al., pigs infected with IA/2014/NADC34 PRRSV had a persistent fever (>40°C) from 3 to 12 dpi and a mortality rate of 14.28% (2 of 14 pigs, died at 9 and 12 dpi, respectively), which demonstrated the high pathogenicity of IA/2014/NADC34 PRRSV (4). Since these two PRRSVs share the high genomic similarity without any recombination with other PRRSV strains, the discrepancy of pathogenicity between U.S. NADC34 PRRSV and Chinese NADC34-like PRRSV could be attributed to the origin of viruses and ages of pigs (3-week-old pigs versus 5-week-old pigs).

To further explore the pathogenicity of Chinese NADC34-like PRRSV, we tested the pathogenicity of JS2021NADC34 PRRSV, a Chinese NADC34-like PRRSV isolated from Jiangsu province of China in 2021 (8). Similar to IA/2014/NADC34 and HLJDZD32-1901 PRRSVs, JS2021NADC34 strain has no recombination with other PRRSVs (data not shown). PAMs infected with JS2021NADC34 PRRSV had apparent CPE at 24 hpi with high virus titers (Fig. 1A to C), which indicated that this strain of virus has adapted well to PAMs and could be highly pathogenic to pigs. As expected, pigs infected with JS2021NADC34 PRRSV had lasting high fever with severely clinical signs, including dehydration, respiratory distress, and shivering. Two of four pigs had moribund conditions at 8 dpi, and one pig died at 10 dpi. Gross and histopathological examination results also supported the high pathogenicity of JS2021NADC34 PRRSV on pigs. Of note, 2-month-old pigs were used in our study, compared to 3-week-old and 5-week-old pigs used in the two above-reported studies, which further revealed the high pathogenicity of JS2021NADC34 PRRSV on older pigs. As mentioned above, both JS2021NADC34 PRRSV and IA/2014/NADC34 PRRSV had higher pathogenicity on pigs than HLJDZD32-1901 did. In addition to considering the difference of pig ages, other factors, including the breed lineage and genetic traits of experimental pigs, could be attributed to the disparity of results. Mixed breed pigs were used in IA/2014/NADC34 PRRSV infection experiments, and Large White-Duroc crossbred PRRSV-free pigs were used in our study. There was no information about pig breed in Song’s study (6).

Several Chinese NADC34-like PRRSVs reported before 2022 were found to have recombination with U.S. PRRSV strains including IA/2014/NADC34, ISU30, and NADC30 (2). Most recently, one Chinese NADC34-like PRRSV was found to have recombination with local PRRSV strain QYYZ, indicating the quick evolution of it to better adapt to domestic pigs (9). In addition to recombination events, patterns of aa deletions different from the 100-aa deletion in NSP2 were also observed, which make NADC34-like PRRSVs more complex in the field (2). Consistent with its genomic changes to have better fitness for domestic pigs, NADC34-like PRRSV has become one of the main epidemic strains in some areas of China (13). Therefore, the characteristics and pathogenicity of NADC34-like PRRSVs warrant further study.

## MATERIALS AND METHODS

### Virus and cells.

JS2021NADC34 PRRSV was isolated as described previously (8). The virus was purified by plaque assay for three rounds. Porcine alveolar macrophages (PAMs) were obtained from 4-week-old specific pathogen-free (SPF) pigs and cultured in RPMI 1640 medium (Gibco BRL Co., Ltd., USA) supplemented with 10% fetal bovine serum at 37°C in 5% CO_2_. The viral supernatants were collected from infected cells at the indicated time points and titrated by TCID_50_ and quantitative reverse transcription PCR (RT-PCR) as described previously (15).

### PRRSV phylogenetic analysis.

For phylogenetic analysis, the ORF5 gene sequences of PRRSV strains in different lineages/sublineages (Table S1) were aligned by MUSCLE using MEGA-X. A phylogenetic tree was constructed using the neighbor-joining method with a bootstrap value of 1,000 replicates.

### Animals and experimental design.

Eight 2-month-old pigs free of pseudorabies virus (PRV), PRRSV, classical swine fever virus (CSFV), and porcine circovirus 2 (PCV2) were randomly divided into two groups with 4 pigs in each group. Pigs in the first group were infected with JS2021NADC34 PRRSV at 3 × 10^6^ TCID_50_/pig via intranasal (0.5 mL/nasal) and intramuscular (2 mL) routes simultaneously. Pigs in the 2nd group received the same volume of DMEM via the same routes as the placebo. Pigs were monitored daily for rectal temperature and clinical signs after viral infection. The pigs were humanely euthanized when they had moribund conditions or at the end of the study, which was terminated at 14 dpi. All animal experiments were approved by the Institutional Animal Care and Use Committee, and conventional animal welfare regulations and standards were taken into account.

### Histopathology and immunohistochemistry staining.

Lung, lymph node, and tonsil samples were collected at necropsy. These samples were fixed in 10% buffered neutral formalin for hematoxylin and eosin and immunohistochemistry staining as described previously (10). The staining was operated automatically by Leica fully automatic dyeing machine. The anti-PRRSV N (4A5) antibody (MEDIAN, Republic of Korea) was used for immunohistochemistry staining. The slides were visualized by 200× microscope photographs.

### Viremia and serological test.

Blood samples of pigs were collected at 0, 3, 7, 9, and 14 dpi for detection of viremia and PRRSV-specific antibody. Total RNA was extracted from serum samples by using an RNeasy minikit (Qiagen, Germany) according to the manufacturer’s instructions. The quantitative PCR (qPCR) was performed as described previously (15).

PRRSV-specific ELISA antibody titers were measured using Herdcheck PRRSV X3 antibody test (IDEXX Laboratories, Westbrook, ME) as described by the manufacturer. PRRSV-specific antibody titer was reported as sample-to-positive (S/P) ratios. The serum samples having an S/P ratio of 0.4 or higher were considered positive.

### Ethical approval.

Animal experimental protocol was approved by the Institutional Animal Care and Use Committee with the reference number 202202001. All animal experiments were performed following relevant guidelines and regulations.

### Data availability.

The data that support the findings of this study are available from the corresponding author upon reasonable request.
