# Supplementary material for: High Pathogenicity of a Chinese NADC34-like PRRSV on Pigs
Source: Microbiol Spectr. 2022 Jun 29;10(4):e01541-22. doi: 10.1128/spectrum.01541-22 (PMC9431460; doi:10.1128/spectrum.01541-22)
Supplement: Supplemental file 1 — Table S1. Download spectrum.01541-22-s0001.pdf, PDF file, 0.1 MB [file spectrum.01541-22-s0001.pdf]

Table 1. PRRSV strains used in this study

PRRSV strains used in this study							
**NO**	**Strains**	**GenBank accession NO**	**Origin**	**NO**	**Strains**	**GenBank accession NO**	**Origin**
1	JS2021NADC34	MZ820388	China	17	JL580	KR706343	China
2	LNWK130	MG913987	China	18	IA/2014/NADC34	MF326985	USA
3	LNWK96	MG860516	China	19	NC/2014/ISU-3	MF326990	USA
4	FJ0908	MK202794	China	20	HLHDZD32-1901	MN648449	China
5	FJZ03	KP860909	China	21	HLJZD22-1812	MN648450	China
6	NB/04	FJ536165	China	22	HLJZD30-1902	MN648055	China
7	CH-1a	AY032626	China	23	LNDZD10-1806	MN648054	China
8	BJ-4	AF331831	China	24	GM2	JN662424	China
9	HUN4	EF635006	China	25	QYYZ	JQ308798	China
10	MN184A	DQ176019	USA	26	ISU30	KT257977	USA
11	VR-2332	AY150564	USA	27	NADC30	JN654459	USA
12	NCV-13	KX192112	USA	28	Em2007	EU262603	China
13	NCV-21	KX192115	USA	29	JXA1	EF112445	China
14	FJFS	KP998476	China	30	JXA1P15	FJ548855	China
15	HENNAN-XINX	KF611905	China	31	CH-2018-NCV-Anheal-1	MH370474	China
16	PRRSV-ZDXYL-China-2018-1	MK453049	China	32	PRRSV-ZDXYL-China-2018-2	MK453050	China
